# Biodegradable magnesium fixation screw for barrier membranes used in guided bone regeneration

**DOI:** 10.1016/j.bioactmat.2021.10.036

**Published:** 2021-12-02

**Authors:** Željka Perić Kačarević, Patrick Rider, Akiva Elad, Drazen Tadic, Daniel Rothamel, Gerrit Sauer, Fabien Bornert, Peter Windisch, Dávid Botond Hangyási, Balint Molnar, Till Kämmerer, Bernhard Hesse, Emely Bortel, Marco Bartosch, Frank Witte

**Affiliations:** a Department of Anatomy Histology, Embryology, Pathology Anatomy and Pathology Histology, Faculty of Dental Medicine and Health, University of Osijek, Osijek, 31000, Croatia; b Department of Prosthodontics, Geriatric Dentistry and Craniomandibular Disorders, Charité – Universitätsmedizin Berlin, Aßmannshauser Straße 4–6, 14197, Berlin, Germany; c Botiss Biomaterials AG, Ullsteinstrasse 108, 12109, Berlin, Germany; d CMF Surgery, Johannes BLA Hospital, Mönchengladbach, Germany; e Dental Clinic, University of Strasbourg, France; f Department of Periodontology, Semmelweis University, Budapest, Hungary; g Xploraytion GmbH, Bismarkstrasse 11, Berlin, Germany; h University Hospital Munich, Department of Dermatology and Allergy, Frauenlobstr. 9-11, 80337, Munich, Germany; i Biotrics Bioimplants AG, Ullsteinstrasse 108, 12109, Berlin, Germany

**Keywords:** GBR, Guided Bone Regeneration, Magnesium, Biodegradable, Implant, GBR, Bone healing, Soft tissue healing

## Abstract

An ideal fixation system for guided bone (GBR) regeneration in oral surgery must fulfil several criteria that includes the provision of adequate mechanical fixation, complete resorption when no longer needed, complete replacement by bone, as well as be biocompatible and have a good clinical manageability. For the first time, a biodegradable magnesium fixation screw made of the magnesium alloy WZM211 with a MgF_2_ coating has been designed and tested to fulfill these criteria. Adequate mechanical fixation was shown for the magnesium fixation screw in several benchtop tests that directly compared the magnesium fixation screw with an equivalent polymeric resorbable device. Results demonstrated slightly superior mechanical properties of the magnesium device in comparison to the polymeric device even after 4 weeks of degradation. Biocompatibility of the magnesium fixation screw was demonstrated in several *in vitro* and *in vivo* tests. Degradation of the magnesium screw was investigated in *in vitro* and *in vivo* tests, where it was found that the screw is resorbed slowly and completely after 52 weeks, providing adequate fixation in the early critical healing phase. Overall, the magnesium fixation screw demonstrates all of the key properties required for an ideal fixation screw of membranes used in guided bone regeneration (GBR) surgeries.

## Highlights

• The first comprehensive report on experimental data for a biodegradable metallic fixation pin for use in oral surgery is presented.
• Results demonstrated superior mechanical properties of the Mg fixation pin in comparison to the polymeric pin even after 4 weeks of degradation.
• The MgF_2_ coated Mg pin made of alloy WZM211 has a slow corrosion rate with a service time of 4 weeks and is fully resorped at 52 weeks after implantation.

## Graphical abstract

**Figure:**
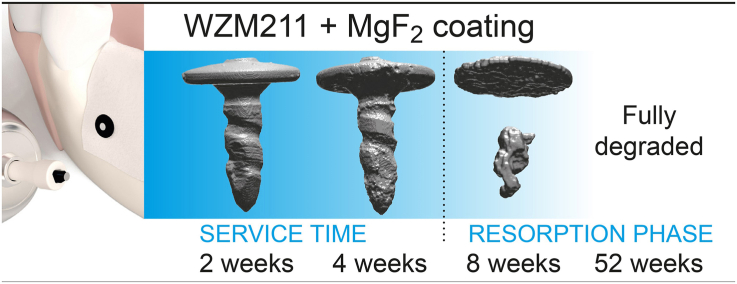

## 1 Introduction

Fixation pins and screws are commonly used in oral surgery to secure barrier membranes for guided bone regeneration (GBR). GBR is a dental surgical technique used for the repair and regeneration of bone and gingival tissue. Membranes can be fixated with a variety of means such as pins, screws or sutures depending on the indication and application site.

The fixation of GBR membranes has been suggested to prevent the transfer of micro movements to the augmented site and the dislocation of the membrane, protecting the underlying blot clot and stabilizing the wound [1]. It has also been shown that for periodontal defects, a rigid fixation of the membrane is crucial for the predictability of reattachment [2].

Currently, the most commonly used fixation systems which are used for either membrane or bone block fixation comprise of either stainless steel [3] or titanium/titanium alloy screws [[3], [4], [5], [6]]. Despite the benefits and proven clinical success of titanium and other metal screws, they also demonstrate some disadvantages. Disadvantages of non-resorbable metal fixation screws include an unacceptable level of palpability, intraoral exposure, passive migration, particle release as well as distortion of magnetic resonance in diagnostic images, making it difficult to visualize the surrounding tissues [[7], [8], [9]]. These impairments are the reason that permanent metal screws should be removed despite their high stability. Biodegradable and resorbable devices therefore have the potential to reduce patient's discomfort and treatment costs [10].

Accordingly, there is a need for resorbable fixation devices in GBR procedures, as most common GBR membranes are already resorbable and their resorbable benefits would be otherwise hampered. Suchenski et al. reviewed available metallic and non-metallic fixation devices and stated that the ideal material for a resorbable fixation system should fulfil three criteria; provision of an adequate mechanical fixation, complete resorption when no longer needed, and complete replacement by bone [11].

Until now, many of the resorbable devices that have been studied are made from biodegradable polymers such as PLGA or PLLA [11]. The use of polymeric resorbable screws has been demonstrated as a viable alternative to the non-resorbable metallic screws [[12], [13], [14]], however they too have reported problems.

Studies have shown that PLLA screws remain easily detectable 24 months after implantation [15]. One study reported the complete degradation of PLLA screws 3 years after implantation, although there were signs of ossification in 81% of the cases [16]. Having a long degradation time of the fixation screw is not necessarily required. The fixation screw only needs to function for as long as the fixated membrane provides a barrier function. Studies on biodegradable collagen membranes have reported visible degradation and collapse of part of the collagen structure between 2 and 6 weeks [17,18]. For instance, in a study with beagle dogs, collagen Bio-Gide membranes showed signs of degradation at the 4-week time point and were almost completely resorbed after 8 weeks [19]. Therefore, after 4 weeks, a functional tissue barrier was no longer provided by the collagen membrane, hence its fixation was no longer required. This sets a minimal functional service time of the magnesium fixation screw to 4 weeks.

Another common complication is the breakage of polymeric fixation screws and has been reported in several studies [14,15,20]. Inflammation and a delayed foreign body reaction up to 12 months post-operatively has also been reported [21], as well as cyst formation [15,22], and an allergic reaction [23]. Another potential concern for using PLLA/PLGA as a material is the risk of osteolysis [20].

Due to the reported complications of resorbable polymeric screws, specifically the tissue response to the degrading screws, alternative technologies have been explored. Magnesium-based devices have proven successful in orthopedic and cardiovascular applications and might provide the necessary properties for a dental fixation screw.

The medical use of magnesium for pins, screws, plates and nails had been suggested as early as 1900 [24]. More recently, magnesium screws based on a MgYREZr(WE43) alloy, have been applied successfully in orthopedics [25] and have proven a viable alternative to titanium [[26], [27], [28], [29]]. However, even though magnesium fixation screws already have been applied to the mandible [30], they have yet to be used within the oral cavity, where many of the benefits observed in orthopedics are expected to be replicated.

The elastic modulus of magnesium is closer to that of bone than that of other metals used for implants; approximately 45 GPa, which is closer to cortical bone (5–23 GPa) than for example titanium (100–125 GPa) [31]. A common issue with the implantation of titanium implants is the production of artefacts during post-operative radiographic analysis [9], however in comparison, magnesium screws generate significantly fewer image artefacts in common imaging modalities of radiography, CT and MRI [32]. Therefore, the use of magnesium screws may facilitate the post-operative follow-up.

Magnesium is an essential element for humans. It has many roles within the body such as being a cofactor for numerous metabolic enzymes [29]. The human body contains between 21 and 28g of elemental Mg in total [33]. About 60% can be found in the bones and 25–30% is stored in the muscles [34]. Magnesium levels within the body are controlled via renal excretion rates, regulated absorption and release and storage of magnesium in the bones [33]. Mg2+ ions can be reabsorbed at the Henle-Schleife (approximately 80% of circulating Magnesium) or can be excreted via the urine [33,34]. Even in instances of chronic renal failure, Mg2+ ions have been shown to be effectively excreted via the intestine in feces [35]. Thus, there is an effective mechanism to eliminate high concentrations of Mg2+ ions if necessary.

Under a normal atmosphere, magnesium metal forms a magnesium oxide layer on its surface, which reacts with humidity to form magnesium hydroxide that deposits on its surface [34]. These layers are susceptible to corrosion, especially in the presence of anions. The main degradation products of magnesium metal under physiological conditions, as determined from immersion corrosion tests using either Hank's balanced salt solution, simulated body fluid or Dulbeccos modified Eagles Medium were MgO, Mg(OH)_2_ and MgCO_3_ [36]. When tested in simulated body fluids, apatites can form on the corroding Mg due to precipitations of calcium and phosphate or carbonates at locally high pH. As a result, there are multiple corrosion processes involved with magnesium under physiological conditions.

As part of the degradation process in an aqueous environment, hydrogen is produced as a by-product in a one to one stoichiometric relation:

(Eq. 1) Mg + 2H_2_O -> Mg^2+^ + 2OH^−^ + H_2_

Based on this chemical reaction, implants made of magnesium can be associated with hydrogen gas accumulations if the initial corrosion rate is too high and more hydrogen gas is produced than the surrounding tissue perfusion can eliminate. Although the gas formation have been found close to moderate inflammation, neither have negatively influenced bone formation [37,38], and the hydrogen gas is known to rapidly diffuse from the site [39]. *In vivo* animal data shows that the initial accumulation of gas is absorbed (e.g. in the fat tissue [40]) or diffused within 2–4 weeks after surgery. Nevertheless, no long-term negative effect of the gas cavity formation has been found in animal studies [41], and spontaneous regression was confirmed in other studies in animals and humans [24,27,28,42,43].

Alloying, surface modifications or conversion coatings can be used to adapt the corrosion rate and hence reduce the rate of hydrogen gas produced, or increase biocompatibility of magnesium implants [44]. Magnesium fluoride surface on magnesium alloys have been shown to reduce hydrogen gas evolution, and are associated with a lower weight loss and volume decrease [45,46]. Magnesium alloy (MgCa0.8) cylinders with and without a magnesium fluoride surface were implanted into the marrow cavity of New Zealand White rabbits [47]. After 6 months of implantation, the cylinders with a magnesium fluoride surface were mechanically stronger than the uncoated cylinders, and had a calcium and phosphorus rich degradation layer on the implant surface.

A magnesium fixation screw has been developed and tested as reported in this article. The magnesium fixation screws, made from a WZM211 alloy, are designed to function similarly to traditional non-resorbable steel or titanium screws, however, have the added benefit of being completely resorbable. The magnesium alloy used for the fixation screw has a magnesium fluoride coating intended to slow down resorption during the first phase of wound healing and thus ensure sufficient stabilization of the barrier membrane.

Tests have been designed to demonstrate the mechanical capabilities, biocompatibility, performance, and safety of this device, thereby proving the functionality of the magnesium fixation screw prior to commencing clinical trials. Mechanical tests of the magnesium fixation screw have compared the device to polymeric fixation screws, as they represent the weakest option of the currently available fixation screws, and therefore provide a baseline for the requirements of the magnesium fixation screw. *In vivo* studies were performed in comparison to titanium fixation screws, which are the most commonly chosen method for membrane fixation in regenerative dentistry and provide a comparison for the expected response from a non-resorbable device (also recommended by ISO 10993–6).

## 2 Materials and methods

### 2.1 Tested material

**Fig. 1:**
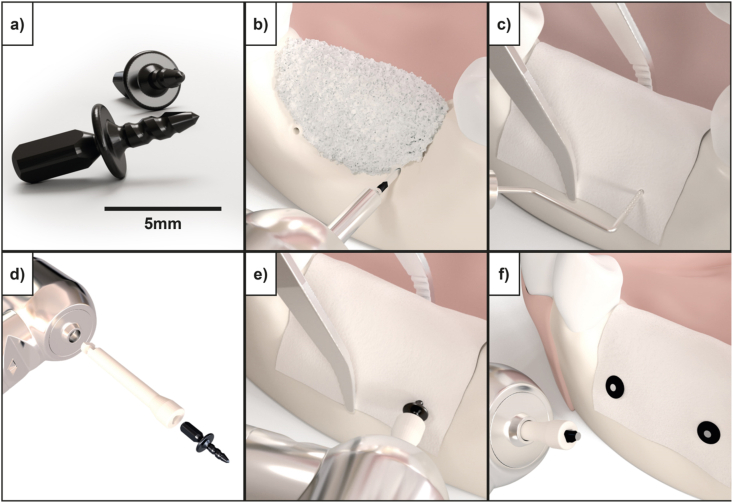
a) Resorbable magnesium fixation screw (NOVAMag® fixation screw XS, botiss biomaterials GmbH) used for securing barrier membranes such as collagen, PTFE as well as pure magnesium membranes in GBR dental surgeries. b-f) Demonstrate the standard fixation protocol for the magnesium screw, in this instance for securing a collagen membrane to the mandible. b) Pilot holes with a 0.9 mm diameter are made for the fixation screw using a precision drill (NOVAMag® precision drill). c) The membrane is placed over the defect and the pilot holes located using a dental probe. d) The fixation screw is attached to a connecting device (NOVAMag® connector) and e) inserted into the pilot holes. f) Once the screw is seated, the drive of the fixation screw will shear off to leave a flat level surface to the screw head.

The magnesium fixation screw (NOVAMag® fixation screw XS, botiss biomaterials GmbH, Germany) used in this study is produced at biotrics bioimplants AG (Berlin, Germany) from the magnesium alloy WZM211 that contains 2 wt% yttrium (Y) 1 wt% zinc (Zn) and ≤1 wt% of manganese (Mn). The screws have a shaft diameter of 1.0 mm, length of 3.5 mm, and a head diameter of 3.0 mm. The average weight of the screw is 17 mg. The screw head extends into a triangular prism shape that is referred to as the “drive” (Fig. 1a). Following a standard insertion protocol as outlined by the manufacturer, the drive is used for transferring torque to the screw during its insertion (Fig. 1e). Once the screw is seated, the drive is sheared off or removed using a pair of pliers (Fig. 1f). The fixation screws are sterilized using gamma irradiation.

During the manufacturing process, the magnesium screws are treated with hydrofluoric acid to produce a corrosion inhibiting MgF_2_ layer on the surface of the screw. In contrast to conventional coatings, no material is applied, but a conversion layer is created by immersion in liquid hydrofluoric acid. Imaging-X-ray photoelectron spectroscopy indicates a homogenous distribution of the elements; magnesium, yttrium and fluorine, although some of the analyzed areas contained yttrium-rich zones (Ø about 50 μm) which were reduced in fluorine concentration.

### 2.2 Preparation to microscopic images

The magnesium samples were embedded in a methyl methacrylate-based resin (Demotec 30, Demotec metallo-graphic, Nidderau, Germany) to reveal the alloys’ microstructure and the morphology and composition of the degradation products layer. After the resin hardened, the surface was ground with silicon carbide paper stepwise down to a grit size of 2500. Then the samples were polished in the presence of water-free oxide polishing suspension (OPS). The residual OPS and the debris was removed in an ultrasonic bath with 100% ethanol. This was followed by optical microscopy and grain size analysis using samples which were etched for a few seconds in picric acid solution dissolved in water (17%), ethanol (79%), glacial acetic acid (4%) (all chemicals by VWR International). Subsequently, the samples were rinsed in 100% ethanol and dried by compressed hot air before optical microscopy.

### 2.3 Sample preparation for WDX and electron microprobe

The magnesium samples were prepared using an argon ion cutting and cross section polisher (JOEL IB-19520 CCP) and were analyzed using an electron microprobe (Jeol JXA-8530F).Amounts of Mg and Fluoride (F) in the films were calculated using the StrataGem film analysis software (v 4.8) based on wavelength dispersive X-ray (WDX) spectra, obtained with a JEOL JXA-8530F electron microprobe at 10 kV.

### 2.4 Initial mechanical properties of tested material

#### 2.4.1 Torque test (torsional yield strength, maximum torque, and breaking angle)

The torque test compared the magnesium fixation screw to an alternative resorbable fixation tacking system that is made of degradable copolymers l-lactide, D,L-lactide, and trimethylene carbonate (Inion GTR™ Tacks). The screws and tacks were embedded in epoxy adhesive (for the magnesium screws: Henkel, Loctite® EA 9514; for the polymeric fixation system: UHU Endfest) in such a way that 20% of the threaded portion and head protruded from the embedding device. A screw nut was bonded to the screw/tack head to grab the specimen for the test and avoid breakup of the drive. The embedded specimens were placed between a screwdriver mounted onto a computer and a torque sensor (Actor, Maxon Motor AG, Type EL45 BL Y 250 KL 2 WE A; Torque transducer, Lorenz Messtechnik GmbH, Type D-2452). The tests were conducted until the samples failed caused by fracture at the embedding level. Six repeats were performed for each group. This test was performed according to the standard ASTM F543-17.

#### 2.4.2 Driving torque test

The driving torque test measured the torque that is required to drive the screw into a standard material. The magnesium fixation screw was compared to an alternative resorbable polymeric fixation system (Inion GTR™ Tacks).

Before commencing the insertion process, test blocks fabricated from solid rigid polyurethane foam, grade 40 (Sawbones Europe AB, Sweden), were prepared with pilot holes according to the individual product insertion protocols. A screw nut was bonded to the screw/tack head to grab the screw during insertion. The samples were then placed between the test block prepared with the pilot holes and a screwdriver that was mounted onto a computer controlled brushless DC motor with a torque sensor (Actor, Maxon Motor AG, Type EL45 BL Y 250 KL 2 WE A; Displacement transducer, Balluff, Type BTL5-A11-M0200-P-S32; Torque transducer, Lorenz Messtechnik GmbH, Type D-2452). The samples were driven into the test block at a rate of 5.0 r/min and the torque and displacement value was measured during complete insertion due to the small specimen geometry. All tests were stopped after complete insertion and was repeated six times per group. This test protocol aligned with the requirements outlined in standard ASTM F543-17.

#### 2.4.3 Pull-out test

The pull-out test measured the axial tensile force which is required for screw/tack failure or for the screw/tack to be extracted from a standard material. The magnesium fixation screw was compared to an alternative resorbable polymeric fixation system (Inion GTR™ Tacks).

**Fig. 2:**
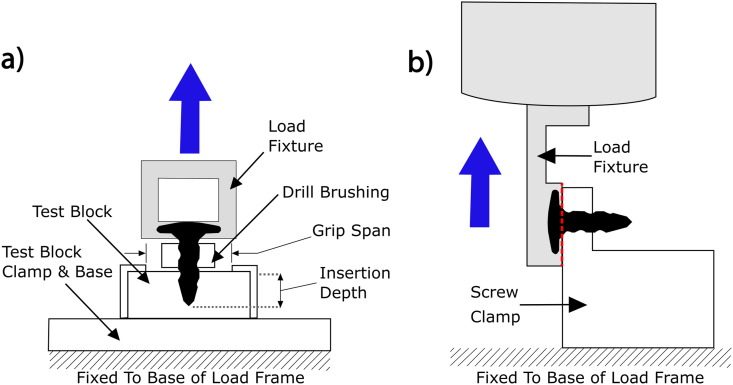
a) Schematic of the “Pull-out” test set-up according to ASTM F543. b) Schematic of the set-up used for the “shear test”. In both schematics, the grey colored pieces of equipment move, whilst the white colored pieces remain in a fixed position.

To this end, the devices were inserted into a solid rigid polyurethane foam, grade 15 (Sawbones Europe AB, Sweden). For the insertion of the magnesium screw, punch marks were used to guide the insertion instead of using pilot holes. Drill bits provided by the manufacturer of the polymeric tacks were used to prepare pilot holes for the insertion the polymeric comparative group. To enable the application of an axial load to the screws/tacks, an M2-screw was bonded with instant glue to the screw/tack heads. A tensile force at a rate of 5 mm/min (Universal testing machine frame, Instron, Type 5544; Force transducer, controlled channel, Instron, Type 2530–437; Displacement transducer, Instron, Type 5544) was used to pull the samples out of the foam block. The set-up for the test is displayed as a schematic in Fig. 2a. The test was stopped upon extraction of the sample from the polyurethane foam block. This test protocol aligns with the requirements outlined in ASTM F543-17.

#### 2.4.4 Surface layer test

The purpose of this analysis was to determine the adhesion and cohesion stability of the coating of the magnesium fixation screw. The adhesion and cohesion of the coating was tested based on a microscopic analysis after a Vickers Indentation on six screws. A diamond indenter (Universal testing machine frame, Instron, Type 5569A; Force transducer, controlled channel, Instron, Type 2525–806; Displacement transducer, Instron, Type 5569A; Vickers Indenter, BAQ, Type Reicherter Briro UVN/Emco) with a square base and a specified angle of 136° between the opposite faces was forced into the surface of the screw. The indentation point was chosen to be on the head of the screw as this was the largest flat surface. A maximum load of 49.03 N was applied. After the indentation, the indentation marks were inspected under a microscope (Digital Microscope Keyence, Type VHX S550E) for indications of induced delamination and fracture of the surface coating.

#### 2.4.5 Surface delamination after screw insertion

The purpose of this analysis was to determine if there was a surface delamination of the coating after screw insertion. The test was repeated six times, with samples visually inspected before and after testing. Screws were inserted following the protocol outlined in section 2.4.2, after which, the samples were removed from the bloc for inspection by cutting open the polyurethane block while preventing direct contact with the screw. The screws were microscopically examined before and after insertion.

### 2.5 In-vitro corrosion tests

#### 2.5.1 Corrosion test set-up

Magnesium screws were degraded for 1, 4, 8, 15, 21 and 30 days in carbonated buffered Hank's Balanced Salt Solution (HBSS) (addition of 0.35 g/l NaHCO_3_). 0.2 mL of solution was used for every 1 mm^2^ surface area of the screw, which is in accordance with standard ASTM G31. Each screw was placed individually in wells filled with 7.8 mL of HBSS and kept in an environmental atmosphere with a relative humidity of 90%, 37 ± 2 °C temperature, and a CO_2_ concentration between 1.0 and 3.0%. The pH of the solution was set at 7.4 and frequently monitored (pH/redox/temperature measuring device with data logger, Greisinger electronic GmbH, Type GMH 3551) and regulated by adapting the environmental CO_2_ concentration. If the measured pH exceeded a pH of ±1, the solution was changed. This occurred only once, on day 16 of the test.

#### 2.5.2 Mass loss

Six groups of five magnesium screws were weighed (High precision balance, Sartorius, Type BP 211-D) prior to testing. The screws were then corroded for a set period of time. After retrieval, the samples were rinsed and immersed in acetone to remove excess electrolyte. After rinsing, the screws were cleaned with chromic acid to remove the corrosion products. The screws were subsequently weighed again and mass loss and corrosion rates calculated.

The average corrosion rate (mm y^−1^) was calculated using equation (2):

(2) CorrosionRate=(K×W)/(A×T×D)

Where $K=8.76\times 10^{4}$, W is mass loss in grams, A is the area of the fixation screw in cm^2^ (0.3926 cm^2^), T is the time of exposure in hours, and D is the material density (1.74 g cm^−3^).

#### 2.5.3 Shear test after corrosion

The purpose of the test was to determine the ability of the magnesium screw to withstand shear loads along the threaded portion of the screw at an initial time point, and after 1, 4, 8, 15, 21 and 30 days of corrosion. Six groups of six magnesium screws were corroded for a set period of time. To set a comparison value, a polymeric fixation tack (Inion GTR™ Tack) was also measured in its non-degraded state.

For shear testing, the specimens were clamped and a tensile shear load was applied (Universal testing machine frame, Instron, Type 5544; Force transducer, controlled channel, Instron, Type 2580–107; Displacement transducer, Instron, Type 5544) approximately 0.4 mm below the head of the screw. The shear load was applied at 1.0 mm/s to the threaded portion of the screw shaft and the longitudinal axis of the screw was aligned perpendicular to the load axis. The test was stopped after failure of the screw occurred. The maximum load applied to the devices was measured. The set-up for the shear test is demonstrated as a schematic in Fig. 2b.

### 2.6 Biological safety assessment

Biological safety was analyzed through multiple cell and animal tests. All animal biocompatibility tests performed for the biological safety evaluation were approved of by the NAMSA Ethical Committee and by the Ministry of Education, Higher Education and Research. Each procedure is part of a project authorization (Authorization numbers 05306.03A and APAFIS#14881–2018021415456720 v2) that is reviewed every five years. Any significant changes to the procedures were approved prior to conduct. NAMSA is an AAALAC international accredited facility and is registered with the French Department of Agriculture for animal housing, care and investigations. All tests were carried out according to the current ISO 10993 series of standards.

#### 2.6.1 Cell media extracts

Eagle Minimum Essential Medium (EMEM) (reference M2279, Sigma-Aldrich), supplemented with 10% (v/v) Fetal bovine serum (FBS) (reference F7524, Sigma-Aldrich), l-glutamine (reference G7513, Sigma-Aldrich) (>2 mM) and antibiotics (2% (v/v) Penecillin (100 units/mL), Streptomycin (>100 μg/mL) (reference P4458, Sigma-Aldrich), and 1% (v/v) Amphotericin B(2.5–3 μg/mL) (reference A2942, Sigma-Aldrich)). Using an extraction ratio of 3 cm^2^ screw surface area to 1 mL of extraction, the magnesium screw was submerged in the extraction media and maintained at a temperature of 37 °C for 72 h. During the extraction process, the extract was continuously agitated. Following extraction, the extract was used immediately for testing. The extract was diluted to concentrations of 50%, 25% and 12.5% (v/v) using EMEM.

In accordance with ISO 10993, a negative, blank, and positive control were prepared. A negative control of a high density polyethylene (HDPE) sheet (Hatano Research Institute, Food and Drug Safety Center Grade) was prepared using an extraction ratio of 6 cm^2^ sample surface area to 1 mL of extraction medium. The negative control was not diluted.

A control blank was prepared the same way as the test article, however without using the magnesium screw. The purpose of the blank control is to assess possible falsifying effects of the extraction vessel, the extraction medium and the extraction process.

A positive control of a polyurethane film containing 0.1% zinc diethyldithiocarbamate (Hatano Research Institute, Food and Drug Safety Center) was prepared using an extraction ratio of 6 cm^2^ sample surface area to 1 mL of extraction medium. The positive control was diluted with EMEM to concentrations of 25%, 20%, 15%, 10% and 3% (v/v).

#### 2.6.2 Polar and apolar extracts

A 0.9% Sodium chloride (SC) (NaCl, CAS No. 7647-14-5) was used as a polar extraction vehicle and sesame oil (SO) (CAS No. 8008-74-0) was used as an apolar extraction vehicle. Additionally, water (WFI) (CAS No. 7732-18-5) was also used as an extraction media. Using an extraction ratio of 3 cm^2^ screw surface area to 1 m#L of extraction media, the magnesium screw was submerged in the extraction media and maintained at a temperature of 50 °C for 72 h. During the extraction process, the extract was continuously agitated. Following extraction, the extracts remained at room temperature and used with 24 h of completing the extraction.

#### 2.6.3 Cytotoxicity

The cytotoxic potential of the magnesium fixation screw was evaluated. Extracts of the magnesium screw, negative control, control blank, and positive control were prepared as described in section 2.6.1.

L-929 mouse fibroblasts in a semi-confluent mono layer were dosed with the extracts and incubated at 37 °C in the presence of 5% CO2 for 24 h. The cells were rinsed three times with Dulbecco's Phosphate Buffered Saline (DPBS) with Ca^2+^ and Mg^2+^ (reference D8662, Sigma-Aldrich) before fresh culture medium was added to the cells. The cells were then incubated with a cell proliferation assay (CellTiter 96® AQueous Non-Radioactive Cell Proliferation Assay with MTS and PMS, reference G5430, Promega) (MTS-PMS) solution before optical density measurements were made with a microplate reader (Tecan, Sunrise, Magellan Standard Version 6.6 software) using a wavelength of 492 nm. The percent viability was determined using the control blank as a reference. If cell viability would have dropped below 70% of the control blank, a cytotoxic potential would have been considered.

#### 2.6.4 Sensitization

The magnesium screw was evaluated for its potential to cause delayed dermal contact sensitization in guinea pigs (young adult, males, *Cavia porcellus*, Dunkin Hartley). SC Polar and SO apolar extracts (see section 2.6.2) were intradermally injected (Induction I) and 6 days later topically applied (Induction II) to 10 test guinea pigs per extract in an attempt to induce delayed sensitization. The extraction vehicles (0.9% saline solution and the sesame oil) without extracts were similarly injected and topically applied to five control guinea pigs (per vehicle). Following a recovery period of 14 days, the challenge was applied, consisting of cotton disks saturated with the test article extract or extraction vehicle, and compressed to the trunk of the animal for 24 ± 2 h. After observational periods of 24 ± 2 h and 48 ± 2 h, each animal was scored according to the Magnusson and Kligman Scale [48].

#### 2.6.5 Irritation or intracutaneous reactivity

The magnesium fixation screw was evaluated for its potential to cause irritation following intracutaneous injections in rabbits (young adult, males, *Oryctolagus cuniculus*, New Zealand White). SC Polar and SO apolar extracts of the magnesium fixation screw were prepared (see section 2.6.2). A 0.2 mL dose of the appropriate test article extract was injected intracutaneously into five separate sites on the back-left side of three rabbits. Similarly, the extract vehicle alone (control blank) was injected on the back-right side of each rabbit. The injection sites were observed immediately after injection. Observations for erythema and edema were conducted at 24, 48 and 72 h after injection and scored according to the severity of the reaction.

#### 2.6.6 Acute systemic toxicity

The magnesium fixation screw was evaluated for acute systemic toxicity in mice (Female, *Mus musculus*, OF1 Ico (IOPS Caw)). WFI and SO extracts of the magnesium fixation screw were prepared (see section 2.6.2). Five mice were given an intraperitoneal injection with a single dose (50 mL per kg of body weight) of the appropriate test article extract. Similarly, a separate group of five mice were dosed with corresponding extraction vehicle alone (blank control). The mice were observed for signs of systemic toxicity immediately after injection and at 4, 24, 48 and 72 h after injection. Body weights were recorded prior to dosing and at 24, 48 and 72 h after injection.

#### 2.6.7 Genotoxicity: mouse lymphoma assay

A Mouse Lymphoma Assay was performed to test for genotoxicity. The Mouse Lymphoma Assay was conducted to evaluate the mutagenic potential of the test article extracts. Using the mouse lymphoma forward mutation assay procedures, mouse lymphoma cells (L5178Y/TK^+/-^ cell line) were exposed to the extracts for a 4 h treatment in the presence and absence of metabolic activation was performed, as well as a 24 h treatment in the absence of metabolic activation.

The test article was extracted in RPMI-1640 serum-free Cell Culture Medium (RPMI_0_) (reference R7638, Sigma-Aldrich) and Dimethyl Sulfoxide (DMSO) (reference D1435, Sigma-Aldrich). The RPMI_0_ extract solution was supplemented with 1% (v/v) l-glutamine (CAS No. 56-85-9), 1.8% (v/v) sodium pyruvate (CAS No. 113-24-6) and 2% (v/v) penicillin-streptomycin (reference P4458, Sigma-Aldrich) and 0.5% (v/v) poloxamer 188 (CAS No. 9003-11-6). Using an extraction ratio of 3 cm^2^ screw surface area to 1 mL of extraction media, the magnesium screw was submerged in the extraction media for 72 h and maintained at a temperature of 37 °C for RPMI_0_ and 50 °C for the DMSO.

Extracts were tested at 8 concentrations: 100%, 50%, 25%, 12.5%, 6.25%, 3.13%, 1.56%, and 0.78% (v/v). The RPMI_0_ extract was supplemented with 5% serum prior to the 4 h and 24 h assessments. The DMSO extract was diluted to a final concentration of 1.0% with RPMI_5_ (RPMI-1640 Cell Culture Medium supplemented with 5% horse serum (reference H1138, Sigma-Aldrich), 1% (v/v) l-glutamine, 1.8% (v/v) sodium pyruvate and 2% (v/v) penicillin-streptomycin and 0.5% (v/v) poloxamer 188) for the 4 h and 24 h assessments.

The magnesium extracts were compared to blank controls (extract vehicle alone) and positive controls. Positive controls of Methyl Methanesulfonate (MMS) (CAS No. 66-27-3) and Cyclophosphamide (CP) (CAS No. 6055-19-2) were used. At the 4 h treatment, MMS was used at a final concentration of 12 and 14 μg/mL, and CP was used as a final concentration of 3 and 3.5 μg/mL. For the 24 h treatment, only MMS was used at a final concentration of 3 and 3.5 μg/mL.

#### 2.6.8 Genotoxicity: bacterial reverse mutation study

A Bacterial Reverse Mutation Study was performed to test for genotoxicity. The bacterial reverse mutation standard plate incorporation study was conducted to evaluate whether extracts of the test article or their metabolites would cause mutagenic changes in *Salmonella typhimurium* tester strains TA98, TA100, TA1535, TA1537 and *Escherichia coli* tester strain WP2*uvr*A (strains purchased from Trinova Biochem, Moltox) in the presence and absence of mammalian metabolic activation. Bacterial reverse mutation tests have been widely used for the determination of mutagenic and potential carcinogenic hazards.

Extract vehicles used were Sodium Chloride (SC) (Reference 600019, Aguettant/Lavoisier) and DMSO using the protocol described in 2.6.2. Tubes containing molten top gar were inoculated with culture from one of the five tester strains, along with the test article extracts with doses of 100 μL/plates for 100%, 50%, 25%, 12.5%, 6.25% and 3.13% (v/v) extracts. An aliquot of phosphate buffer or rat liver S9 Mixture (reference 11-01L, Trinova Biochem, Moltox) providing metabolic activation was added. The mixture was poured across the triplicate plates. Parallel testing was conducted with control blanks and positive controls. The mean number of revertants for the test extract plates was compared to the mean number of revertants of the appropriate control blank plates for each of the five tester strains.

### 2.7 *In-vivo* corrosion kinetics study on pigs

This study was performed to evaluate the degradation kinetics of subperiosteally placed magnesium fixation screws in combination with a collagen membrane on the buccal side of the mandible using Yucatan minipigs. Animals were observed for signs of dehiscences and gas cavities. Nine animals were used in this study. Samples were collected at three time points (2 weeks ± 3 days, 4 weeks ± 3 days and 8 weeks ± 3 days post-implantation) and analyzed using synchrotron-radiation based microtomography (SR-μCT).

#### 2.7.1 Animal model

The Yucatan minipig study has been performed at BRIDGE PTS, Texas, USA. The protocol was reviewed and approved by the Testing Facility's Institutional Animal Care and Use Committee (IACUC). The review ensured compliance with Title 9 Code of Federal Regulations, “Animals and Animal Products,” Chapter 1, Subchapter A, “Animal Welfare,” Parts 1, 2, and 3, as well as the “Guide for the Care and Use of Laboratory Animals” published by the National Research Council. The Testing Facility is accredited by the Association for Assessment and Accreditation of Laboratory Animal Care (AAALAC) and has received its Domestic Assurance certification OLAW: # A4672-01.

#### 2.7.2 Design of corrosion kinetics study

**Fig. 3:**
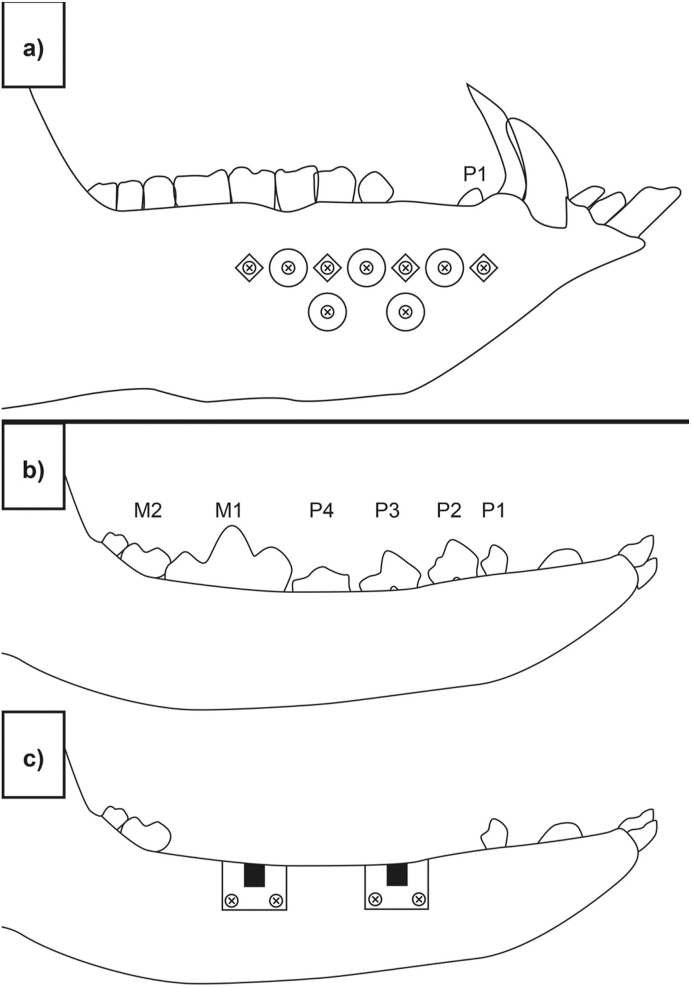
Approximate positions of the defect/implant sites used for the *in vivo* studies. a) Subperiosteally placed magnesium fixation screws in combination with a collagen membrane on the buccal side of the mandible of Yucatan minipigs. For each animal, half of the mandible was implanted with five magnesium fixation screws fixating circular sections of a collagen membrane (Bio-Gide, Geistlich). The position of each magnesium screw was marked by non-resorbable titanium screws (1.5 mm × 3 mm ProFix titanium screws, Osteogenics) The titanium screws are represented by screw heads in a diamond shape. The images b), c) and d) represent the surgical intervention performed on Beagle dogs in the *in vivo* performance study. b) In a preparatory surgery, four teeth between the mandibular second premolar to the first molar (PM2 to M1) on each side of the lower jaw and the corresponding teeth of the upper jaw were surgically extracted. c) After a healing period of 12 ± 2 weeks, two independent bone defects were created on each side of the lower jaw. The defects were filled with bone substitute material and covered with collagen membrane fixed with either 4 titanium or magnesium fixation screws (2 on buccal and 2 on lingual side). d) For histological evaluation, sections were taken from the central region of the defect (orange area) and from the position of the fixation screw (blue area).

Nine Yucatan minipigs (*Sus scrofa*) were used in the study; three per time point. On each animal, the surgeon created a 3–4 cm long incision approximately 8 mm below the teeth (beginning at the canine). A full subperiosteal-gingival flap was raised by carefully lifting the periosteum from the underlying bone using an elevator or similar instrument. For each animal, half of the mandible was implanted with the magnesium fixation screw and circular sections of a collagen membrane (Bio-Gide, Geistlich). Five collagen membrane samples were individually fixed with one magnesium fixation screw per sample on one side of the mandible. The position of each magnesium screw was marked by non-resorbable titanium screws (1.5 mm × 3 mm ProFix titanium screws, Osteogenics) which were inserted into the mandible, as demonstrated in Fig. 3a. The flaps were closed hermetically using traditional surgical technique and with interrupted suturing.

All locatable samples were removed upon termination (2 weeks ± 3 days, 4 weeks ± 3 days and 8 weeks ± 3 days post-implantation). Samples were removed and isolated with at least 1 mm of the surrounding tissue from the adjacent implants via a 10.0 mm internal diameter trephine burr. The samples were then fixed and stored in 100% ethanol.

#### 2.7.3 Micro computed tomography

Screw degradation was measured via volumetric changes recorded using synchrotron-radiation based microtomography (SRμCT). SRμCT data was collected from the tissue samples containing the magnesium screw using the SRμCT setup beamline PSICHE and ANATOMIX at the Synchrotron SOLEIL facility in Saint-Aubin, France. The experimental conditions at PSICHE were set as follows: The X-ray beam energy was set to 45 keV (pink beam). The optics were set such that the isotropic voxel size resulted in 3.0 μm. For each scan, the number of projections was automatically adjusted to obtain the optimal resolution as function of dose and diameter of the field of view. Acquisitions were collected in half-acquisition mode over 360°. Acquisition time per projection was 50 ms.

For the experimental conditions at beamline ANATOMIX, the energy of the beam (pink-beam) was set to 35 keV and pixel size was set to 3.25 μm. The number of projections per tomographic scan was automatically adjusted as above. To cover the full length of every screw inside the resulting fields of view in either beamline, 1–3 scans of each sample were collected in different z-positions with ∼20% overlap between them. The data were reconstructed using Paganin's method. The reconstructed volumes were stored in 16-bit format. The diameter of each 3D data set ranged from 2500 to 4000 pixels; the height ranged from 2000 to 6000 pixel.

To quantify the morphology, the screw had to be segmented from other objects (such as bone, air, and sample holder) present in the CT volume. Evaluation of the corrosion of the magnesium fixation screw over time was achieved by measuring and comparing the device volume and surface area.

#### 2.7.4 Statistical analysis

Mean, standard deviation and statistical significance between time points (unpaired t-tests) were performed using GraphPad Prism 8.1.2 software.

### 2.8 *In-vivo* performance study on dogs

This GLP study was designed to evaluate the safety and efficacy of the magnesium fixation system screw compared to the titanium screw (ProFix Screw, Osteogenics) control to fix a collagen membrane (Bio-Gide, Geistlich) placed over 3-wall defects filled with bone substitute material (Bio-Oss, Geistlich) in healed extraction sites using a canine mandibular defect model. Comparisons were made at an early time point (1 week: 7 ± 1 day), intermediate time point (8 weeks: 56 ± 5 days) and late time point (16 weeks: 112 ± 5 days) post-implantation. Eighteen (18) canines were enrolled in this study plus two (2) spare animals as backup also underwent surgical teeth extraction and implantation. These spare animals were then transferred to an additional 52 week cohort. The study has been performed at AccelLab, Quebec, Canada.

#### 2.8.1 Animals model

The dog model represents a fully functional *in vivo* anatomical model for bone healing evaluation following defect creation and bone remodeling [[49], [50], [51], [52]]. The mandibular defect model enables an assessment and comparison of the local tissue effects and the performance of the screws. Adult dogs have a similar bone density to humans therefore, canine bones are representative of the implantation of human implants and prostheses. Canine bone tissue exhibits similar mechanical properties, morphological structures and healing capacity to human bone. In addition, dog bones are also large enough to allow multiple experimental procedures [[49], [50], [51], [52]]. As this study evaluates the healing process following a surgery, in-vitro or computer-generated models cannot be used.

The protocol was reviewed and approved by the Testing Facility's Institutional Animal Care and Use Committee (IACUC). The review ensured compliance with Canadian Council on Animal Care (CCAC) regulations. The Testing Facility is accredited by the Association for Assessment and Accreditation of Laboratory Animal Care (AAALAC) and the CCAC.

The minimum number of animals possible was utilized in the study. A total of 18 animals +2 spares were enrolled on the study. A group size of 6 animals per time point was used.

#### 2.8.2 Design of the performance study

The procedure was carried out in two phases: A preparatory phase and an experimental phase.

In the preparatory phase, four teeth between the mandibular second premolar to the first molar (PM2 to M1) on each side of the lower jaw and the corresponding teeth of the upper jaw were surgically extracted (Fig. 3b), followed by wound closure of the upper jaw only. A healing period of 12 ± 2 weeks followed with suture removal at 2 ± 1 week post extractions, and daily oral cavity flushing for 10–14 days post-extractions. Wound site follow-up evaluation took place twice during the first week post-extraction, with or without anesthesia, followed by once a week thereafter until wound sites were fully healed.

In the experimental phase, a second surgery was performed. Two independent bone defects were created on each side of the lower jaw only. The defects were filled with bone substitute material (Bio-Oss, Geistlich) and covered with collagen membrane (Bio-Gide, Geistlich). The membranes were either fixed with 4 titanium screws (ProFix Screw, Osteogenics), 2 on the buccal and 2 on the lingual side, as a control, or with 4 magnesium fixation screws, also with 2 on the buccal and 2 on the lingual side. Each animal received a total of 4 defect sites implanted with a total of 16 screws (i.e., 8 magnesium fixation screws for the test group, and 8 titanium fixation screws for the control group). A schematic for the defect site and implant placement is depicted in Fig. 3c. This was followed by wound closure. Suture removal took place at about 2 ± 1 weeks post implantation and daily oral cavity flushing for 10–14 days post-implantation.

Over the course of the study, the animal health status was followed by a veterinarian team, with significant observations such as swelling and wound dehiscence reported and monitored. This is important for evaluating the soft tissue healing response to the magnesium fixation screw.

#### 2.8.3 Histology processing

Non-decalcified histology was performed. The bone defects were separated and individual blocks containing the implant and surrounding soft and hard tissues were embedded in methylmethacrylate (MMA) resin. The polymerized MMA blocks were sectioned in a bucco-lingual plane into two pieces. At least two lingual-buccal sections were made from each defect site (targeting the center of the defect) and stained with H&E and Goldner's Trichrome (GT). Several additional slides were generated, focusing on the position of the four magnesium or titanium fixation screws, and were stained with H&E. The center of the defect is defined as the area augmented with the bone substitute material that is covered with the collagen membrane. The screws are positioned either side of the defect, and are used for fixating the collagen membrane into the animal's native bone. Sections taken from the central region of the defect were made to determine if the corrosion process of the magnesium screw influenced the tissue response within the defect, whereas section taken from the positions of the screws were used for evaluating the direct material/tissue interaction (Fig. 3d).

Golden trichrome (GT) stained sections taken from the central region of each defect site were digitally captured to obtain whole-section images, which were then analyzed using Image-Pro Premier 9.2 or higher software to obtain histomorphometric data of interest. Histomorphometric measurements of the GT stained sections evaluated bone regeneration by measuring new bone growth, soft tissue infiltration, hemorrhage, and the amount of void space from the center of the defect. These parameters were graded according to a numerical scale (0 = Absent, 1 = Minimal, 2 = Mild, 3 = Moderate, 4 = Marked).

Pathological parameters of the H&E and GT stained histology sections were analyzed and graded according to cell type and responses, as listed in Table 1. The H&E slides were used to assess the inflammatory response, necrosis, neovascularization, fibrosis, fatty infiltrate, fibrinous exudates, tissue degeneration, particulate debris, evidence of membrane, soft tissue infiltration, hemorrhage, and void parameters, within the central region of the defect as well at the position of the fixation screws. The GT stained slides were used to assess the new bone growth, fibrosis, presence of the membrane, soft tissue infiltration, bone resorption, and void space parameters, only for the central region of the defect. The data collected for the central region of the defect was combined for the H&E and GT stained sections.

**Table 1:** Histological grading according to the cell type and tissue response, adapted from ISO 10993-6.

Response	Score *(phf = per high powered (x400) field)				
	0	1	2	3	4
Polymorphonuclear cells	0	Rare, 1–5/phf*	6-10/phf	Heavy infiltrate	Packed
Lymphocytes	0	Rare, 1–5/phf	6-10/phf	Heavy infiltrate	Packed
Plasma cells	0	Rare, 1–5/phf	6-10/phf	Heavy infiltrate	Packed
Macrophages	0	Rare, 1–5/phf	6-10/phf	Heavy infiltrate	Packed
Giant cells	0	Rare, 1–2/phf	3-5/phf	Heavy infiltrate	Packed
Necrosis	0	Minimal	Mild	Moderate	Marked
Fibrinous exudates	0	Minimal	Mild	Moderate	Marked
Tissue degeneration	0	Minimal	Mild	Moderate	Marked
Neovascularization	0	Minimal capillary proliferation focal, 1–3 buds	Groups of 4–7 capillaries with supporting fibroblastic structures	Broad band of capillaries with supporting structures	Extensive band of capillaries with supporting fibroblastic structures
Fibrocytes/fibroconnective tissue, fibrosis	0	Narrow band	Moderately thick band	Thick band	Extensive band
Fatty infiltrate	0	Minimal amount of fat associated with fibrosis	Several layers of fat and fibrosis	Elongated and broad accumulation of fat cells about the implant site	Extensive fat surrounding the implant

Selected values were expressed as group means ± standard deviation per cohort and individual values. Statistical analyses of selected parameters were conducted using SigmaPlot software to compare test and control group within time-points.

## 3 Results

### 3.1 Microstructure and coating

**Fig. 4:**
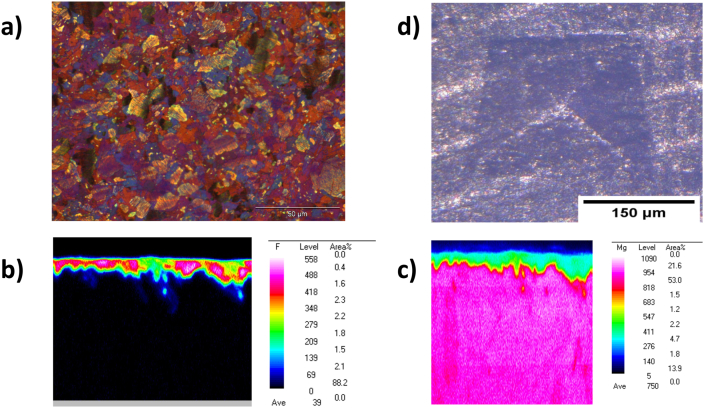
(a) Polarized optical overview image of the Mg screw's microstructure showing. The size of the globular grains is about 6 μm with a range of 3–25 μm. (b) Fluoride mapping with WDX demonstrates that there is a continuous MgF_2_ coating on the screw. (c) Mg mapping with WDX confirms that Mg is mainly in the bulk material and in smaller amounts in the coating layer. (d) The Vickers hardness test indicates that there is no delamination and heavy cracks of the coating at edges of the deformation, indicating a strong adhesion of the MgF_2_ coating to the bulk material.

The optical microstructure of the WZM211 alloy demonstrates a globular grain with a wide size range between 3 and 25 μm. The average grain size was found to be about 6 μm. The grains contained several Long-Period Stacking Order (LPSO) phases which are known to provide a high strength material (Fig. 4a) [53]. The magnesium fluoride coating seems to provide a continuous covering over the bulk metal alloy WZM211 as shown in Fig. 4b and c.

### 3.2 Mechanical tests

Multiple mechanical tests were performed comparing the magnesium and polymeric fixation devices. These results are summarized in Table 2. A torque test was performed on the devices to establish the maximum torque that they could withstand. The mean torsional strength for the magnesium screw was 0.033 ± 0.001 Nm compared to 0.0004 ± 0.0001 Nm for the polymeric device. The mean maximum breaking torque was 0.043 ± 0.001 Nm and the mean breaking angel was 36 ± 4° for the magnesium fixation screw, however these parameters could not be determined for the polymeric alternative.

**Table 2:** Biomechanical properties of magnesium and polymeric fixation devices.

Material properties	Magnesium fixation screw	Polymeric fixation tack
Maximum Torque [Nm]	0.043 ± 0.001	Not measurable
Breaking angle of drive [°]	36 ± 4	Not measurable
Insertion torque [Nm]	0.0128 ± 0.0042	0.0056 ± 0.0012
Insertion depth [mm]	3.2 ± 0.3	3.0 ± 0.1
Maximum axial load [N]	6.83 ± 0.90	0.77 ± 0.40
Tensile shear load [N]	117 ± 22	33 ± 2

To compare how these results translate to the application of each device, a driving torque test was performed that measured the insertion torque required to insert each device when following the standard insertion protocols as outlined by each manufacturer. The mean insertion torque was 0.0128 ± 0.0042 Nm and the mean insertion depth was 3.2 ± 0.3 mm for magnesium device compared to 0.0056 ± 0.0012 Nm mean insertion torque and 3.0 ± 0.1 mm mean insertion depth for the polymeric device. During insertion, none of the magnesium screws failed, however 2 of the polymeric screws fractured.

A pull-out test was performed to measure the axial tensile force that is required for the fixation device to fail (fracture/break) or for the device to be extracted. During the test, no failures of the magnesium nor polymeric devices occurred. The mean maximum load applied to the devices for their extraction from the material was 6.83 ± 0.90 N for the magnesium screw and 0.77 ± 0.40 N for the polymeric device.

The adhesion and cohesion of the magnesium fluoride coating of the magnesium screw was tested using microscopic analysis after a Vickers Indentation test. The microscopic images of the indentation marks did not indicate the occurrence of cracks in the surface or surface delamination, as demonstrated in Fig. 4d. Hence, no obvious damage to the surface layer was observed. Furthermore, no cracks at the outer edges of the indentation were observed for any of the samples, which indicates that the coating has a high resistance against mechanical forces.

For the surface delamination test of the magnesium screw the mean value for the insertion torque was 0.0103 ± 0.0022 Nm and the screws inserted to an average depth of 4.2 ± 0.6 mm. The tested devices were microscopically examined before and after and showed no signs of failures.

### 3.3 In-vitro corrosion

The *in-vitro* corrosion test demonstrated a gradual increase in corrosion rate, before reaching a peak of 1.12 ± 0.18 mm/y after 15 days of corrosion (Table 3). Between 15 and 30 days, the corrosion rate decreased, but remained higher than that measured over the initial 8 days of corrosion.

**Table 3:** Immersion corrosion test results for the magnesium fixation screw.

Degradation time [d]	Weight loss [mg]	Weight loss per day [mg/d]	Corrosion rate [mm/y]
1	0.04 ± 0.02	0.04	0.18 ± 0.08
4	0.36 ± 0.05	0.09	0.46 ± 0.07
8	0.98 ± 0.12	0.12	0.63 ± 0.08
15	3.53 ± 0.53	0.24	1.12 ± 0.18
21	3.97 ± 0.25	0.19	0.98 ± 0.06
30	4.43 ± 0.35	0.15	0.76 ± 0.06

Shear tests on the magnesium fixation screws were performed at every timepoint of the corrosion study. The initial shear resistance of the uncorroded magnesium screw was compared to that of a polymeric resorbable fixation system (Table 4). The magnesium fixation screw withstood higher shear loads than the polymeric alternative in an uncorroded state. After 30 days under corrosive conditions, the maximum shear load of the magnesium fixation screw remained higher (approximately 3 times larger) than that of the undegraded polymeric device.

**Table 4:** Shear test after degradation for the magnesium fixation screw.

Degradation time [d]	maximum load applied to the magnesium fixation screw [N]	maximum load applied to the polymeric tack [N]
0 (Initial)	117 ± 22	33 ± 2
1	135 ± 8	not determined
4	136 ± 9	not determined
8	116 ± 16	not determined
15	118 ± 5	not determined
21	108 ± 7	not determined
30	98 ± 18	not determined

### 3.4 Biological safety assessment

The biological safety of the magnesium screw was analyzed using cytotoxicity, sensitization, irritation and intracutaneous reactivity, acute systemic toxicity, and genotoxicity tests (summarized in Table 5).

**Table 5:** Biocompatibility tests results for the magnesium fixation screw.

Biocompatibility Test	Result	Outcome
Cytotoxicity	No cytotoxic effects were observed after incubation of L-929 mouse fibroblasts with the magnesium screw extract up to the highest test concentration of 100%.	Is not cytotoxic
Sensitization	Neither polar nor apolar extracts induced a delayed sensitization in a guinea pig model.	Does not cause sensitization
Irritation or intracutaneous reactivity	Compared to the controls, the polar and apolar extracts had similar reports of erythema and edema in rabbits.	Does not cause irritation or intracutaneous reactivity
Acute system toxicity	Magnesium screw extracts injected into mice to determine acute systemic toxicity did not result in any mortality nor evidence of systemic toxicity.	Does not cause acute systemic toxicity
Genotoxicity	Mouse lymphoma assay: Mouse lymphoma cells showed no mutagenicity when exposed to extracts with concentrations between 0.78 and 100% (v/v). Bacterial reverse mutation study: No mutagenic or genotoxic activity was caused by the magnesium fixation screw extract on the bacterial tester strains.	Is not genotoxic

Cytotoxicity was evaluated via an *in-vitro* study, by measuring the metabolic activity and the proliferation of L929 mouse fibroblast cells in contact with extracts from the magnesium fixation screw. No cytotoxic effects were observed after incubation of the cells with the magnesium screw extract up to the highest test concentration of 100%. Microscopic evaluation did not reveal any cytotoxicity based on cell seeding errors, growth characteristics or change of cell morphology. Thus, the magnesium fixation screw can be considered non-cytotoxic.

Polar and apolar extracts were used to determine sensitization reactions using a guinea pig model (Magnusson-Kligman test). Neither extract (SC or SO) induced a delayed sensitization in the guinea pig model. Based on these results, the magnesium fixation screw was not considered to be a sensitizer.

Polar and apolar extracts were also used to determine irritation and intracutaneous reactivity. When compared to the controls, the magnesium fixation screw extracts had similar observational outcomes of erythema and edema. Therefore, the magnesium screw is not considered to cause irritation or intracutaneous reactivity.

Magnesium screw extracts injected into mice to determine acute systemic toxicity did not result in any mortality or evidence of systemic toxicity. Therefore, the magnesium fixation screw does not cause acute systemic toxicity.

For the mouse lymphoma assay testing for genotoxicity, both the RPMI_0_ and DMSO extracts with concentrations between 0.78 and 100% (v/v) showed no mutagenicity. Moreover, no mutagenic or genotoxic activity was caused by the magnesium fixation screw extract used in the bacterial reverse mutation study. Therefore, the magnesium fixation screw is not considered genotoxic.

### 3.5 In-vivo corrosion kinetics study

**Fig. 5:**
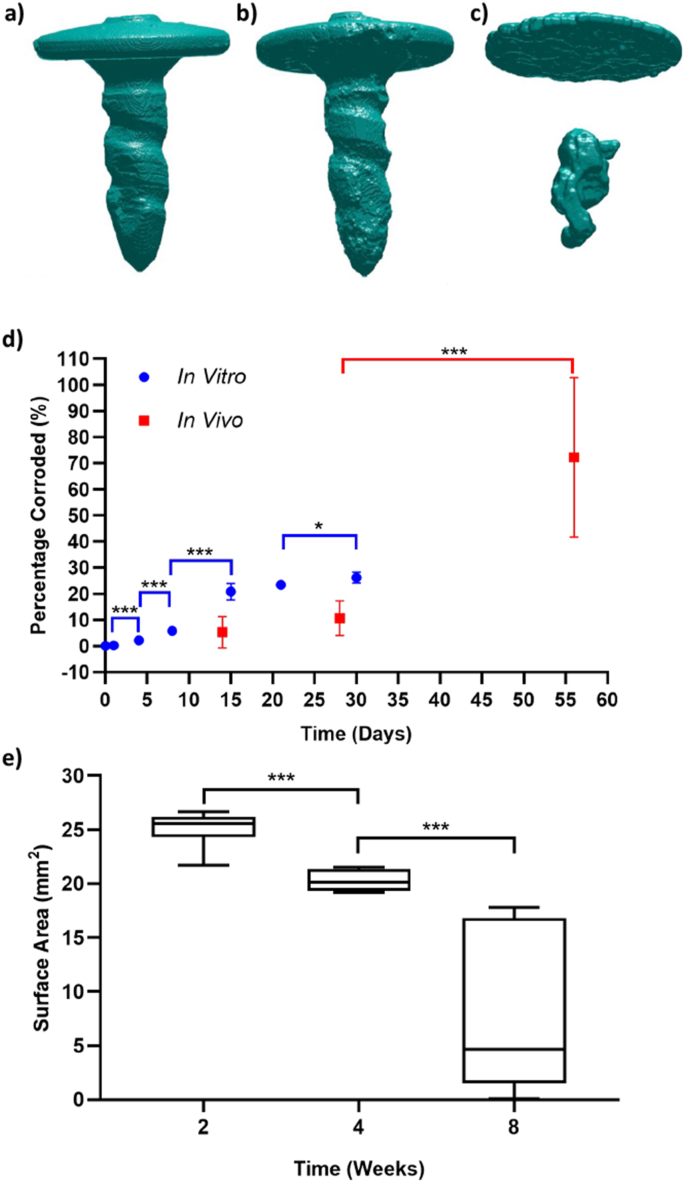
a-c) 3D images of the magnesium screw corroding after implantation into Yucatan minipigs after, a) 2 weeks, b) 4 weeks, and c) 8 weeks. 3D renderings were reconstructed using μCT data of explants. d) Percentage of the magnesium fixation screw corroded during immersion corrosion testing (blue circles) and after implantation in Yucatan minipigs (red squares). e) Boxplot of the mean and standard deviation for the surface area of magnesium fixation screw at the different time points of the *in vivo* corrosion study. In d) and e), a * indicates p < 0.05, and *** indicates p < 0.001.

Representative images of the segmented screw for the three different implantation times are shown in Fig. 5a–c. The magnesium screw gradually corrodes, although its shape remains largely intact for the first 4 weeks.

In several incidences, the minipigs experienced wound dehiscence unrelated to the implanted device, which caused an increase in corrosion rate. Therefore, the data from these individual minipigs was not included for calculating the screw corrosion shown in Fig. 5d and e. Data from 2 pigs was used for the 2 week time point, all 3 pigs for the 4 week time point, and 1 pig for the 8 week time point.

Over the initial 4 weeks after implantation, the magnesium screw volume remained stable (Fig. 5d). It can be seen that the total volume normalized by the initial screw volume remains relatively high and non-significantly different (P = 0.0826) for the first two time points (2 weeks, 4 weeks), with an average volume and standard deviation of 95 ± 6% and 89 ± 6.5% respectively. At the final time point (8 weeks) the screw volume dropped to 27 ± 30.5%, significantly lower than the volume measured at 4 weeks (P < 0.001).

Although the volume of the screw was stable over the initial 4 weeks after implantation, the surface area was significantly different between week 2 and week 4 (P < 0.001) (Fig. 5e). The surface area continued to decrease up to week 8 and was also significantly lower than the surface area at week 4 (P = 0.0006). At the 8 week time point, there is a large spread in the values of volume and surface area, indicating that the individual corrosion rates of the magnesium screws differed past the 4 week time point.

### 3.6 In vivo performance study on beagle dogs

#### 3.6.1 Histological evaluation

**Fig. 6:**
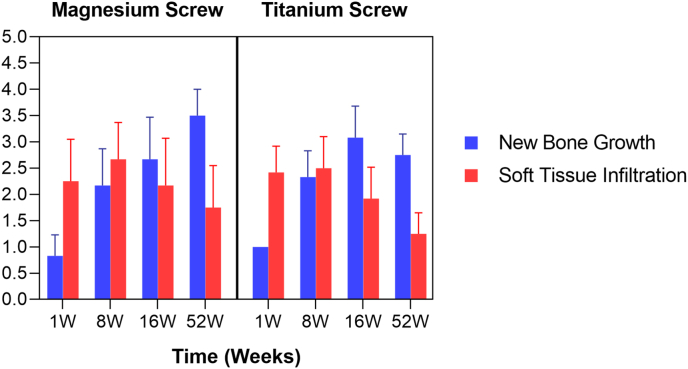
New Bone Growth and soft issue infiltration in the central defect.

Within the central region of the defect sites, the amount of new bone growth was on average greater for the titanium screw group (1.00, 2.33, and 3.08) compared to the magnesium screw group (0.83, 2.17, and 2.67) at 1, 8, and 16 weeks, respectively, with the average scores increasing over time (Fig. 6.). However, at 52 weeks, the trend was reversed and the average new bone growth score was greater in magnesium screw group (3.50) compared to titanium screw group (2.75), with magnesium screw group having two scores of 3 and two scores of 4, whilst the titanium screw group had one score of 2 and three scores of 3.

Soft tissue invasion into the central defect was slightly greater in the titanium screw group (2.42) compared to the magnesium screw group (2.25) at 1 week, with the trend reversing at later timepoints. At 8, 16, and 52 weeks, the magnesium group had a greater average score (2.67, 2.17, and 1.75, respectively) compared to the titanium group (2.50, 1.92, and 1.25, respectively).

Hemorrhage within the defect was evident only at the early (1 week) timepoint, with a slightly greater average score (1.17) occurring in the magnesium screw group compared to the titanium screw group (0.75). Hemorrhage was not evident at later timepoints. When present, the hemorrhage was presumably due to the surgical procedure.

The size of the void within the defect decreased over time, with the void evident in the magnesium group at 1, 8, and 16 weeks (average scores of 1.75, 1.08, and 0.83, respectively), whilst void space was only evident in the titanium group at 1 week (average score 0.50). In the titanium group, voids were caused by processing artefacts (i.e. shrinkage of tissue, resulting in clear space), and the size of each void was always minimal (score 1). The size of the voids in the magnesium group ranged from minimal to moderate (scores of 1–3) at 1 and 8 weeks, and was minimal, mild, or marked (scores of 1, 2, or 4) at 16 weeks. The voids were mostly located surrounding the biodegrading magnesium-based screws, but voids also extended away from the screw sites and into the center of the defect, resulting in the scores noted above. Notably, all voids were resolved by 52 weeks due to the advanced stage of bioresorption that had occurred in the magnesium group.

**Fig. 7:**
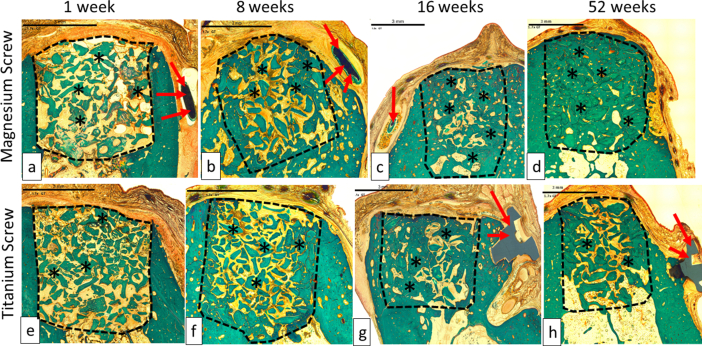
Representable scanned Goldner's Trichrome histology images of GBR performance study on beagles. Dotted Line = edges of the defect site; Asterisks (*) = particles of bone filler material within the defect site; Red Arrow = control and/or test articles; (a), (b), (c) and (d) are presenting a magnesium screw which is degrading over time, and by 16 weeks (c), only small residual particles of the magnesium screw are left, surrounded by new bone. The images (e), (f), (g) and (h) are presenting a control screw made of titanium, which was visible at all time points; 1 week (e), 8 weeks (f), 16 weeks (g) and 52 weeks (h). In each image, the scale bar represents 3 mm.

**Fig. 8:**
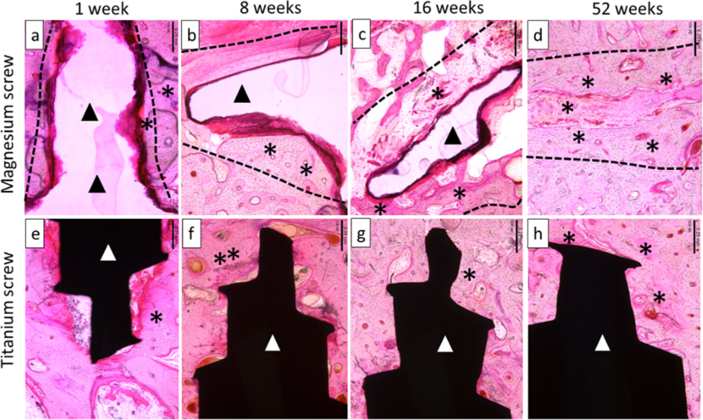
Representable scanned hemalaun eozin histology images of GBR performance study on beagles. Dotted Line = edges of the defect site where the screw was implanted; asterisks (*) = particles of new bone around screws. Black Ars = magnesium screw; (a), (b), (c) and (d) are present the magnesium screw where we can see that is degrading/reabsorbing over time. At 52 weeks (d), the magnesium screw is completely absorbed and replaced by the formation of new bone. White Ars = titanium screw; (e–h) present the titanium screw at all time points 1 week (e), 8 weeks (f), 16 weeks (g) and 52 weeks (h). In each image, the scale bar represents 0,25 mm.

The collagen membrane or its remnants were visible in all sites at 1 week, in most sites at 8 and 16 weeks, and in only 1 out of 4 sites at 52 weeks in the titanium group, with no visible membrane in the 4 sites at 52 weeks in magnesium group (Fig. 7 and Fig. 8.)

#### 3.6.2 Tissue response

Post-implantation, the regular veterinarian monitoring reported few instances of swelling, with no prevalence of occurrence in either test group during the first week post-surgery. Later instances of swelling were slightly more likely to occur in the magnesium group (8/40 treated sites) compared to the titanium group (2/40 treated sites). Neither group indicated the presence of a chronic inflammation reaction such as prolonged redness, swelling, pain and loss of function.

##### 3.6.2.1 Within the defect

The inflammation within the central region of the dental defects was greater in the magnesium screw group compared to the titanium screw group at 1, 8, 16, and 52 weeks, with the greatest difference occurring at 8 weeks. For the first 16 weeks, the inflammation in both groups was mostly associated with the membrane, but by 52 weeks, this had changed to the bone substitute material.

The inflammatory response was mixed with neutrophils, lymphocytes, plasma cells, giant cells, and increased macrophages in the magnesium group at 8 weeks, while the inflammation was limited to macrophages and giant cells in the titanium group. By 16 weeks, the inflammation response was remined similarly composed in the magnesium group. In the titanium group at 16 weeks, the response included occasional lymphocytes and plasma cells along with the macrophages and giant cells. By 52 weeks, the mixed nature of the inflammation in the magnesium group had resolved, and both treatment groups had evidence of macrophages and few multinucleated giant cells, however there was a slightly greater average score for each cell type occurring in magnesium group compared to titanium group. No evidence of active necrosis of the alveolar bone was found at 1, 8, 16, or 52 weeks in either treatment group.

Within the defect, neovascularization was similar across treatment groups, with all sites having a score of 1.00 after the first week, and a score of 2.00 thereafter.

The average fibrosis score was similar for both treatment groups, with an average score of 1.00 for after 1 week; a slightly greater average score for the magnesium group at 8 weeks (2.17) compared to the titanium group (2.08); and equivalent average scores for both groups at 16 and 52 weeks (2.00). The fibrosis was scored based on the thickness of the fibrous connective tissue near the surface of the defect, which included the area of the membrane. Thus, fibrosis within the defect increased from 1 to 8 weeks, and then leveled out to a consistent score of 2 (moderately thick band) at 16 and 52 weeks.

Fibrinous exudates were evident only at the early timepoint (1 week); this appeared as areas of fibrin near the surface of the defect (beneath the membrane) and the average score was similar for both groups (1.75 for the magnesium group and 1.50 for the titanium group).

Fatty infiltrate, tissue degeneration, and particulate debris were not observed in any defect site at 1, 8, 16, or 52 weeks.

##### 3.6.2.2 Surrounding the fixation screw

The inflammation surrounding the magnesium fixation screw and titanium screw was greater for the magnesium screw at all timepoints. The average score for the magnesium fixation screw group increased up to 16 weeks, whereas for the titanium screw, inflammation decreased between 1 week and 52 weeks. By 16 weeks, the average score for inflammation surrounding the magnesium screw was scored as a 3.00 compared to 1.33 for the titanium group, and at 52 weeks the average score remained as 3.00 in the magnesium group, but had slightly reduced in the titanium group (1.25).

The inflammation around magnesium fixation screws was composed of a mixed inflammatory cell infiltrate through 16 weeks, with numerous neutrophils and macrophages at 1 week, and with the addition of lymphocytes, plasma cells, and giant cells at 8 and 16 weeks. The greatest average inflammatory cell score at 16 weeks was for macrophages (average 3.00), with fewer giant cells (1.83), plasma cells (1.33), lymphocytes (1.17), and polymorphonuclear cells (neutrophils, 0.50). The macrophages, lymphocytes, and neutrophils occurred in the fibrovascular tissue that surrounded the biodegrading screw remnants, and the giant cells were numerous within the tissue that surrounded the shaft of the screw and extended into the marrow spaces within the pre-existing alveolar bone. The giant cells contained what appeared to be particulate debris (brown-grey intracytoplasmic material) in all sites at 8 and 16 weeks (presumably biodegradation products from the degradation of the magnesium-based device). Lymphocytes and plasma cells were interspersed throughout the areas of giant cells within the marrow spaces; this was presumably due to the chronic antigenic stimulus from the degradation products.

## 4 Discussion

The magnesium fixation screw is designed specifically for the fixation of barrier membranes used in GBR during oral surgery (Fig. 1). To determine its fixation capabilities, the fixation screw underwent benchtop tests. To provide a reference for the requirements of the screw regarding its mechanical properties, it was compared to an alternative polymeric resorbable fixation device.

Bone anchorage of the fixation screw was evaluated using a pull-out test that demonstrated that the magnesium fixation screw required a force approximately 9 times larger than the comparable polymeric device to be removed after its insertion. Fixation stability, which in this instance is defined as a resistance to shear forces and hence the ability to maintain the original position of the fixated membrane, was evaluated using shear tests. The maximal shear load that the magnesium fixation screw could resist was approximately 3.5 times greater than that of the polymeric device.

Therefore, the mechanical properties of the magnesium fixation screw are superior to that of the polymeric device, and is suitable for the fixation of membranes. Further benchtop tests performed on the magnesium screw demonstrated that it could be successfully inserted without damaging the screw or its protective magnesium fluoride coating. Additionally, the coating was analyzed after the screw had been indented and showed no signs of delamination or cracking, corroborating the stability and cohesion of the surface coating and the bulk material.

As the magnesium fixation screw is resorbable, it is important that it maintains a secure fixation of the membrane during the critical healing period that is required to seclude the bony defect from fast growing gingival epithelial and connective tissues. *In-vivo* studies indicate that resorbable collagen barrier membranes provide a functional barrier for an estimated 4 weeks. A study on collagen membranes in a GBR model in beagle dogs performed by Rothamel et al. showed signs of degradation at 4 weeks post-implantation and were almost completely resorbed after 8 weeks [19]. This rate of collagen membrane degradation is supported by other animal studies performed in rats and rabbits [17,19]. Therefore, the magnesium fixation screw should maintain its fixation function for the duration of the membrane barrier function.

To assess the degradative behavior of the fixation screw and its influence on the screw's mechanical properties, immersion corrosion tests were performed. Over a 30 day period under corrosive conditions, the fixation screws were measured at regular intervals for their mass and maximum shear load. At every time point (1, 4, 8, 15, 21 and 30 days), the magnesium fixation screw remained stronger than the initial strength of the polymeric device (Table 4).

Due to the limitations of performing mechanical tests on fixation screws *in-vivo* after implantation, the data collected from mechanical tests after the immersion corrosion test in-vitro provide the basis of the mechanical stability assumptions made during *in-vivo* tests. Using Yucatan minipigs, the *in-vivo* degradation of the magnesium screws was established.

After 4 weeks degrading, the fixation screws largely maintained their initial shape and volume (Fig. 5). The 4 week *in-vivo* time point corresponds to an equivalent level of corrosion as measured between 8 and 15 days during the immersion *in-vitro* corrosion test. Based on the assumption that the mechanical properties of the screws are equivalent due to similar degrees of corrosion; at 4-week *in-vivo*, the magnesium fixation screws should withstand a maximum shear load between 116 N and 118 N. Hence, the magnesium fixation screw remains stronger than the initial strength of the polymeric device and therefore should maintain a secure fixation over the initial 4-week post-implantation period required to secure the membrane as indicated by Rothamel et al. [19].

The correlation of magnesium corrosion rates between *in vitro* and *in vivo* data has been established to have a ratio between 1 and 4.9 [54]. This published ratio aligns with equivalent corrosion rates measured for the magnesium fixation screw in this study.

Yet, the comparison between *in vitro* and *in vivo* corrosion rates to human clinical data has not yet been established. However, by using published studies, it is possible to make an estimation on this ratio. An *in vivo* study using magnesium alloy screws (MgYREZr) that were implanted into the marrow cavity of the left femora of New Zealand White rabbits demonstrated a complete degradation of the screw over a 12-month period [55]. The same magnesium screw (MgYREZr) has been found in several human clinical studies to be stable for an initial 6–12 weeks and completely corroded within 1–3 years [25]. Therefore, the factor between the corrosion rates in humans and animals, as indicated by these reported studies, is expected to be between 1 and 3.

With a corrosion rate ratio for *in vivo* (animal studies) and human clinical data between 1 and 3, it is expected that the magnesium fixation screw in this study will provide a secure fixation during the critical healing period in human clinical applications. It is also expected that the fixation screws will securely fixate the membrane whilst it provides a barrier function.

Although the magnesium screws had not completely corroded during the duration of the Yucatan minipig study, the screws substantially corroded between the 4 and 8 week time points. Potentially, over the first 4 weeks after implantation, the magnesium fluoride coating protected the underlying WZM211 magnesium alloy from corroding, which is supported by the study performed by Wolters et al. [45]. After 4 weeks, the magnesium fluoride coating was dissolved, enabling a more rapid corrosion of the underlying magnesium alloy. Additionally, as the screw corroded, its surface area increased. As magnesium corrosion is a surface reaction [56], the corrosion rate should continue to increase as the surface area enlarges. As the final time point of the minipig study was 8 weeks, it is expected that the increased corrosion rate would continue until the remaining magnesium screw completely corroded.

The preclinical performance study in beagle dogs directly compared the clinical outcome of GBR defects treated using bovine bone granules and a collagen membrane that had been fixated with either magnesium or titanium screws. The soft tissue response reported by the veterinarian, demonstrated a slight increase in instances of swelling in relation to magnesium screws. This was not considered an indicator for an underlying infection as it is only one of the four clinical signs used to indicate infection: 1. “Rubor” (redness), 2.”Calor” (elevated local temperature), 3. “Dolor” (pain), 4. “Tumor” (swelling). All animals behaved normally and had no observable local redness, nor pain or elevated temperature at the postoperative wound checks, therefore did not indicate an underlying inflammation, which was also confirmed by histological examination. Thus, the temporarily slightly enhanced swelling during the initial phase after implantation can be more likely linked to either local gas formation and/or enhanced local perfusion due to the locally released magnesium ions and/or hydrogen [37,38].

Inflammation investigated by histopathologic analysis found that overall, the inflammation within the central region of the defects was on average slightly greater in the magnesium group compared to the control group at 1, 8, 16 and 52 weeks. A mild response was on average reported for the magnesium group at 1, 8, and 16 weeks, compared to a mild/minimal response at the same timepoints for the titanium screw. The number of inflammatory cells around the screw was also greater in magnesium group compared to control group at all time points. The inflammation in the magnesium group was composed of a mixed inflammatory cell infiltrate, with numerous neutrophils and macrophages at 1 week, and an addition of lymphocytes, plasma cells, and giant cells at 8 and 16 weeks. The macrophages, lymphocytes, and neutrophils occurred in the fibrovascular tissue that surrounded the biodegrading screw remnants, and the giant cells were numerous within the tissue that surrounded the shaft of the screw. The giant cells contained what appeared to be particulate debris in all sites at 8 and 16 weeks (presumably biodegradation products from the degradation of the magnesium-based device, literature suggests amorphous calcium phosphate [57]). At 52 weeks, the inflammatory cell score included only macrophages and single giant cells. The head of the magnesium fixation screw was typically separated from the screw shaft due to the ongoing corrosion process. The shaft of the magnesium screw appeared to be fragmented with mild local inflammation that was restricted to its implantation bed and surrounded the small corrosion fragments at 52 weeks. However, since the inflammatory cell population converted from a mixed inflammatory response at 16 weeks to a homogeneous macrophage and multinucleated giant cell response at 52 weeks, signified the transition from a mixed inflammatory response associated with M1-type macrophages (i.e. pro-inflammatory response) to a more benign response associated with M2-type macrophages (an anti-inflammatory, pro-resolution response) around magnesium screws. This response has been similarly reported in a paper discussing the pathology of bioresorbable implants in other preclinical studies [58].

Many giant cells containing possible magnesium degradation products were evident in the magnesium group, an expected response already known from other magnesium implant materials [59]. The nature of the inflammation signified that the biodegradation products were not eliciting an ongoing reaction in the surrounding tissues and the inflammation did not interfere with the bony regeneration of the surrounding alveolar bone. When this process was compared to a biostable, titanium-based screw, the biostable screw behaved biologically as expected with low-level inflammation. Nevertheless, as the expected higher inflammation associated with the magnesium fixation screw did not result in less newly formed bone, it can be concluded that the device is safe and performs as intended with comparable inflammation in the defect center.

The veterinarian report stated that slightly more postoperative swellings for the magnesium fixation screw group. However, no signs of irritation nor an increase in overall inflammation were noted. Additionally, the swelling did not lead to wound dehiscence or visible signs of (chronic) pain in the animals. Therefore, the swelling is not expected to become an issue when the screws are used in a human clinical setting.

Neovascularization was shown to be present within the connective tissue that had grown into the dental defect sites. Neovascularization was similar in all samples with a score of 1 at week 1 and a score of 2 in week 8, 16 and 52 for both treatment groups. It is known that magnesium ions can locally enhance the neovascularization [60], however in this case the released magnesium ion dosage seemed to be below the effect level to promote more vascularization compared to the control. It can be concluded that both fixation screws allowed new blood vessel formation, an important feature in tissue regeneration as neovascularization provides sufficient supply of blood and regenerative cells to the defect area [61].

The findings in the histopathological analysis regarding the healing response demonstrated that there were no statistical differences in new bone growth, soft tissue infiltration and hemorrhage between the two groups in all four analyzed time points. Therefore, it can be concluded that the fixation capabilities of the magnesium fixation screw have enabled the collagen membrane to remain in place for the duration of the membrane's functional lifespan. However, unlike the titanium screws that may have to be removed in a second surgical procedure, the magnesium screws are resorbable and do not require surgical removal.

Nevertheless, the magnesium metal corrodes faster than the speed of the advancing front of new bone growth. The space between the newly developing bone and the metallic magnesium is occupied by magnesium salts, by-products of the magnesium corrosion process and have disappeared after 16 weeks. During the early phase of degradation, magnesium implants produce corrosion products, for instance, magnesium reacts with water, is hydroxylated to form magnesium hydroxide [Mg(OH)_2_], magnesium chloride [MgCl_2_] and hydrogen gas [62]. The hydrogen gas production results in voids surrounded by a thin fibrous capsule, which is completely resolved by 52 weeks [63]. As shown by histomorphometric analysis, these gas cavities had no negative impact on tissue healing. The formation of gas cavities is to be expected, as it is reported by previously published studies using magnesium implants. However, as indicated by these reports and collaborated by this study, the formation of gas cavities is only temporary and does not have a negative effect on bone regeneration [24,27,28,42,43].

Therefore, it can be concluded, that both fixation systems made either of magnesium or titanium do not negatively impact the bone healing response in the central bone defect and fixes sufficiently the barrier membranes. Furthermore, the degradation process of magnesium fixation screw shows no negative impact on the clinical outcome.

## 5 Conclusion

An ideal fixation system must fulfil several criteria that includes the provision of adequate mechanical fixation, complete resorption when no longer needed, complete replacement by bone, as well as biocompatibility and clinical manageability [11]. Adequate mechanical fixation was shown in several benchtop tests that directly compared the magnesium fixation screw with an equivalent polymeric resorbable device. Results demonstrated even superior mechanical properties of the magnesium device in comparison to the polymeric device. Biocompatibility of the magnesium fixation screw was demonstrated in several *in vitro* and *in vivo* tests, concluding that the device can be safely used. Degradation of the magnesium screw was investigated in *in vitro* and *in vivo* tests, where it was found that the screw is resorbed slowly, providing adequate fixation in the early critical healing phase with a more rapid corrosion profile thereafter. The faster degradation of the device after fulfilling its fixation function leads to temporarily occurring gas cavities that are filled progressively with newly formed bone. Overall, the magnesium fixation screw demonstrates all of the key properties required for an ideal membrane fixation screw used in GBR surgeries. Therefore, it might provide a valid and safe alternative to titanium or resorbable polymeric screws.

## Ethical statement

We further confirm that any aspect of the work covered in this manuscript that has involved either experimental animals or human patients has been conducted with the ethical approval of all relevant bodies and that such approvals are acknowledged within the manuscript.

## Data availability statement

Data are available from the authors with the permission of Botiss Medical AG and Biotrics Bioimplants AG. The data that support the findings of this study are available from the corresponding author, FW, upon reasonable request.

## Disclosure statement

PR and ZP are employees of botiss biomaterials GmbH and FW and MB are employees of biotrics bioimplants AG.

## Funding sources

This research did not receive any specific grant from funding agencies in the public, commercial, or not-for-profit sectors.

## CRediT authorship contribution statement

**Željka Perić Kačarević:** first author, Writing – original draft. **Patrick Rider:** equivalent first author, Writing – original draft. **Akiva Elad:** Methodology. **Drazen Tadic:** Methodology, Funding acquisition. **Daniel Rothamel:** Methodology, Funding acquisition. **Gerrit Sauer:** Methodology. **Fabien Bornert:** Methodology. **Peter Windisch:** Methodology. **Dávid Botond Hangyási:** Methodology. **Balint Molnar:** Methodology. **Till Kämmerer:** Methodology. **Bernhard Hesse:** Methodology. **Emely Bortel:** Methodology. **Marco Bartosch:** Methodology. **Frank Witte:** Conceptualization, Supervision, Methodology, Writing – original draft.

## Declaration of competing interest

The authors declare the following financial interests/personal relationships which may be considered as potential competing interests:The following authors are employees of the company biotrics bioimplants AG (Frank Witte, Marco Bartosch) and botiss biomedical AG (Zeljka Peric Kacarevic, Patrick Rider, Drazen Tadic) which companies have financed the study.A CE mark has been successfully applied for the biodegradable magnesium barrier membrane using the published data in this manuscript.
